# Deep Learning-Assisted Analysis of GO-Reinforcing Effects on the Interfacial Transition Zone of CWRB

**DOI:** 10.3390/ma17235926

**Published:** 2024-12-04

**Authors:** Jiajian Yu, Zhiwei Chen, Xiaoli Xu, Xinjie Su, Shuai Liang, Yanchao Wang, Junqing Hong, Shaofeng Zhang

**Affiliations:** School of Transportation and Civil Engineering, Nantong University, Nantong 226019, China; 2333310013@stmail.ntu.edu.cn (J.Y.); 2433320041@stmail.ntu.edu.cn (Z.C.); xuxiaoli@ntu.edu.cn (X.X.); 2133110318@stmail.ntu.edu.cn (X.S.); 2233110393@stmail.ntu.edu.cn (S.L.); yanchaowang1989@ntu.edu.cn (Y.W.); hongjq@ntu.edu.cn (J.H.)

**Keywords:** graphene oxide, cemented waste rock backfill, nano-modification, pore structure, pore throat, reinforcing feature characterization

## Abstract

Understanding the enhancing mechanisms of graphene oxide (GO) on the pore structure characteristics in the interfacial transition zone (ITZ) plays a crucial role in cemented waste rock backfill (CWRB) nanoreinforcement. In the present work, an innovative method based on metal intrusion techniques, backscattered electron (BSE) images, and deep learning is proposed to analyze the micro/nanoscale characteristics of microstructures in the GO-enhanced ITZ. The results showed that the addition of GO reduced the interpore connectivity and the porosity at different pore throats by 53.5–53.8%. GO promotes hydration reaction in the ITZ region; reduces pore circularity, solidity, and aspect ratio; enhances the mechanical strength of CWRB; and reduces transport performance to form a dense microstructure in the ITZ. Deep learning-based analyses were then proposed to classify and recognize BSE image features, with a high average recognition accuracy of 95.8%. After that, the deep Taylor decomposition (DTD) algorithm successfully located the enhanced features of graphene oxide modification in the ITZ. The calculation and verification of the typical pore optimization area of the location show that the optimization efficiency reaches 9.6–9.8%. This study not only demonstrated the deepening of the enhancement effect of GO on the pore structure in cement composites and provided new insights for the structural modification application of GO but also revealed the application prospect of GO in the strengthening of CWRB composites and solid waste recycling.

## 1. Introduction

Cemented waste rock backfill (CWRB) plays an important role in underground engineering construction [1]. It alleviates the shortage of cement raw materials and improves the ecological environment. It can also improve the utilization rate of resources and provide economic benefits. The waste rock backfill technology with a reasonable ratio can provide cost-effective and environmentally friendly building materials for underground engineering [2]. However, the main reason why CWRB is difficult to widely use in practical underground engineering is that it does not have excellent mechanical properties and good durability [3]. If backfill materials cannot meet the expected requirements of the project, it increases the support demand, reduces the efficiency of mining, leads to uncontrolled ground pressure, and endangers the safe operation of underground projects. The interfacial transition zone (ITZ) has an important impact on the performance of CWRB and is also a weak area in CWRB [4]. ITZ is located at the junction of cement slurry and aggregate and is a complex heterogeneous region with different physical and mechanical properties. Due to the large number of unhydrated cement particles, the volume fraction of hydration products is small and the porosity is high. This affects the interface between cement slurry and aggregate, causing ITZ poor mechanical properties and making it easy to become the origin point of cracks. Therefore, the ITZ plays an important role in the performance evaluation of the whole material at the macro level. The ideal ITZ can significantly improve the mechanical properties, durability, and stability of CWRB [5]. On the contrary, if the ITZ performance is poor, it may lead to strength loss, stress concentration, erosion resistance, and stability reduction, which will affect the performance of the entire backfill system [6]. Therefore, strengthening the microscopic characteristics of the ITZ defect area in CWRB is very important for promoting the application of CWRB and the safety and stability of underground engineering.

In recent years, nanoreinforced cement has been a hot topic in the area of new building materials [7,8] due to its excellent physical and mechanical properties, ultra-high specific surface area, abundant oxygen-containing functional groups, and small dosage ratio. Graphene oxide (GO) has opened up a new way of high-performance cementing composite materials [9]. Gao et al. modified CWRB with 0.007 wt% industrial GO and fly ash, reducing cement consumption by 20% while improving mechanical properties and impermeability by 16.9–38.9% [1]. Cheng et al. found that GO nanosheets significantly improved the microscopic pore structure of CWRB, reducing the total porosity by 31.2%. With the help of GO, the transfer between internal pores changes from a large equivalent pore size distribution to a small equivalent pore size distribution [10,11]. Sun et al. studied the effect of GO on the microstructure of cement mortar ITZ [12]. They found that the incorporation of GO significantly reduced the thickness and porosity of the ITZ, improved the compressive strength, and made the overall microstructure of mortar more uniform. Lu et al. [13] enhanced the ITZ of RFA by coating recycled fine aggregate (RFA) particles with GO, increasing the 28-day compressive and bending strength of recycled mortar by approximately 25% and 20% [13]. In addition, water absorption and chloride diffusion coefficients were reduced by about 20% and 27% [13]. This series of research results shows that GO can improve the mechanical properties of cement-based materials, enhance durability, and optimize the ITZ [14]. Many scholars have proposed the potential application effects of GO-reinforced gelling composites [15,16]. Therefore, the ITZ with GO-modified CWRB in this study has a significant enhancement effect and application prospects [10].

As a recycled aggregate, high water absorption and porosity of CWRB often lead to ITZ damage, making it the most vulnerable part of the concrete composite [1]. These related findings suggest that GO has the potential to enhance the ITZ of CWRB. However, understanding the microstructure modification mechanism of GO is a prerequisite for optimizing the enhancement benefits of GO for the ITZ. Conceptually, GO is a nearly perfect two-dimensional material that is very soft and rich in surface oxygen-containing functional groups [17]. Since the oxygen-containing functional groups in GO can absorb water molecules, thus providing a precursor for the hydration reaction of cement, the hydration degree of cement can be improved [18]. At the same time, it can offer more nucleation sites for ITZ, exert its excellent pore-filling and bridging effect, form hydrogen bonds with a hydroxyl group (-OH) on the surface of calcium silicate hydrate (C-S-H) and calcium hydroxide (C-H), and improve the hydration degree of the ITZ in CWRB [16,19]. Therefore, the introduction of GO into cement-based composites opens up a new way, and it is of great significance in effectively improving the mechanical properties and durability of CWRB by strengthening the micropore structure of the ITZ through GO [16].

Here, we pioneered an image learning analysis method to study the pore enhancement effect of the ITZ after GO modification and analyze the pore structure feature model. Image deep learning analysis has the advantages of automation and efficiency, accuracy and consistency, big data processing ability, and potential advantages in feature extraction and classification, optimization design, and improvement. In this study, we used the metal intrusion method and BSE image to study the mechanism of GO’s influence on pore size distribution, porosity, and pore throat connectivity in the ITZ. Then, the circularity, solidity, and aspect ratio of the pore profile in the ITZ are studied. Through the study of these pore parameters, the enhancement benefits and pore throat characteristics of GO in the ITZ are systematically discussed. After obtaining ideal recognition accuracy, the convolutional neural network (CNN) algorithm is used to extract the pore structure model of the representation precision map with deep Taylor decomposition (DTD). Finally, the model of extracting typical feature regions is analyzed, the pore deterioration region of ITZ is successfully located, and the optimization degree of GO is revealed.

## 2. Method

### 2.1. Sample Preparation

The preparation process of GO-modified CWRB mainly includes two main steps: gangue aggregate mixing and grout preparation. The coal gangue waste used in this study came from coal mining, and its particle size was divided into 7 grades of 0–0.5 mm, 0.5–1.0 mm, 1.0–1.5 mm, 1.5–2.5 mm, 2.5–5 mm, 5–8 mm, and 8–10 mm for screening [20,21]. Talbot gradation theory (TGT) calculates the distribution of coal gangue gradation [20,21]. According to previous research reports, when the Talbot grading index reaches 0.6, the cemented gangue fill has dense microstructure and ideal mechanical properties and impermeability [1,22]. To further study and obtain the microscopic pore structure with excellent permeability resistance and durability, the Talbot grading index of 0.6 was used to configure gangue particles. The literature [20] proved that the strengthening effect of GO on cement-based materials was balanced with the material cost when the mass fraction of GO and polycarboxylate superplasticizer (PC) in dispersions was 0.08 wt% and 0.64 wt% [23], respectively. First, the GO and PC powders were accurately weighed and ultrasonically dispersed at a power setting of 150w to obtain a highly dispersed GO suspension. Based on previous correlation studies [22], the variable range of the water-to-cement ratio was selected to be between 0.4 and0.7. The main reason was to balance the working performance, strength, durability, economy, and construction convenience of cement-based materials. In this range, cement-based materials have the best overall properties and can meet the requirements of most engineering applications. Therefore, the prepared suspension was added to the mixture of cement and fly ash at the water-to-cement (W/C) ratios of 0.4, 0.5, 0.6, and 0.7 respectively (cement mass fraction was 80%, FA mass fraction was 20%) to obtain the GO group [22]. To highlight the enhancement effect of GO at different w/c ratios, a control group (Ref) was obtained by mixing the same amount of water and PC with pure cement at the same w/c ratio [22]. During the experiment, the mass ratio of slurry to gangue aggregate was set at 0.35. Before pouring the CWRB sample, the gangue particles of Talbot 0.6 were accurately weighed, poured into the mixer, and stirred at 180 RPM for 3 min to ensure the uniformity of the stone. The slurry was then poured into the aggregate and stirring continued for 3 min at high (200 rpm/min) and medium (100 rpm/min) speeds [21]. Finally, the fresh mixture was poured into a cylindrical mold with a radius of 50 mm and a height of 100 mm and shaken with a shaker to remove air from the gelled material and ensure that the matrix was dense. The curing conditions ensured a suitable rate of cement hydration, provided sufficient moisture, and prevented cracking while complying with international standards and engineering practices. CWRB specimens were placed in a curing box with a constant temperature of 20 ± 5 °C and 90% humidity for 28 days [21].

### 2.2. Microstructure Characterization

To study the microscopic pore enhancement effect of GO on the ITZ in CWRB at different w/c ratios, the modification characteristics of GO were identified and located, the sample was cut for a metal intrusion test, and the microscopic properties of cement-based materials were analyzed using the captured BSE images. The detailed operations were as follows. After 28 days of curing, the samples were cut to obtain several cube samples with 5mm side lengths and soaked in ethanol solution to prevent further hydration reaction of the samples [24]. The samples were dried in an oven at 105 ± 2 °C for 24 h before metal intrusion, and then, the pores were filled with field metal in the field [25]. The optical contrast between the CWRB matrix and the pore wall was enhanced by metal intrusion into the pores, and the morphology and distribution of the pores became more obvious [26]. BSE imaging technology can better capture the fine features of the material and enhance the visibility of the pores [27]. Before electron microscope observation, the samples were polished with 80, 240, 400, 600, 1000, and 2500 grade sandpaper, and polished with 8000, 15,000, 30,000, and 80,000 grade polishing paste. The purpose of grinding and polishing was not only to make the surface of the CWRB sample flat and smooth but also to improve image contrast and sharpness. More importantly, it helped reduce the damage and background noise of the electron beam on the sample so that the microstructure of the cement-based material could be accurately displayed, such as the distribution of hydration products and pore structure characteristics. Finally, the polished sample was observed under a scanning electron microscope. Before characterization, a two-nanometer-thick gold film was sprayed onto the polished cube surface to reduce charge interference during characterization, and the scanning electron and residence time were set to 5 eV and 10 µs, respectively. The magnification of the BSE image was unified to 400 times, and the pixel size was set to 1280 × 960 pixels [28]. More details about the metal intrusion treatment and BSE observation process can be found in the literature.

To study the nanoscale modification characteristics of GO on the ITZ in CWRB and visualize the enhancement effect of GO on the ITZ, we propose an innovative deep learning method based on BSE images. Previous reports have demonstrated that machine learning has significant advantages in analyzing the microscopic properties of cement-based materials from an image perspective [29,30], mainly due to automation and high efficiency. Deep learning models such as convolutional neural networks (CNN) can automatically extract complex image features and perform high-precision recognition and classification [31]. Junlin et al. conducted a comprehensive analysis based on BSE images and studied the microstructure of ordinary Portland cement (OPC) and silica-reinforced OPC from four perspectives: qualitative visualization, quantitative physical descriptor, statistical correlation function, and deep learning interpretation [30]. Image analysis provides a new method to characterize the microstructure of nanoreinforced cement composites.

## 3. Results and Discussion

### 3.1. Effect of GO on the Pore Size Distribution of CWRB Samples Under Different W/C Ratios

**Figure 1:**
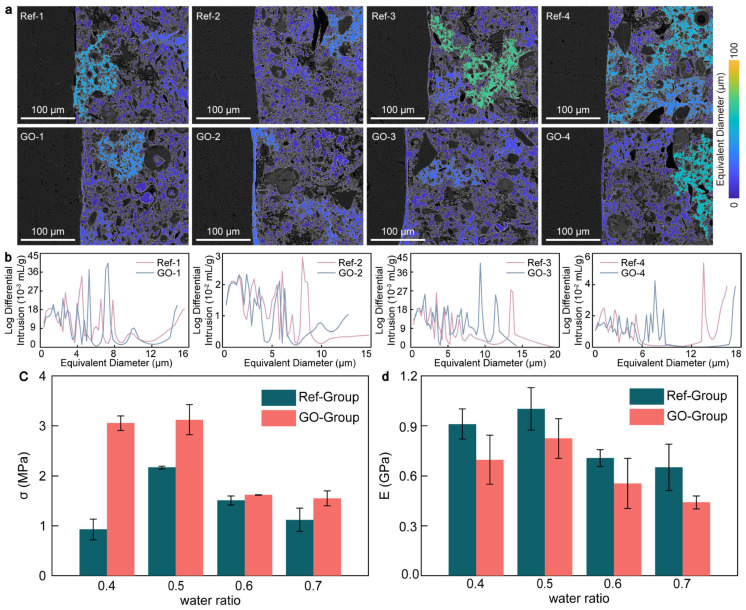
(**a**) ITZ aperture color map of CWRB sample; (**b**) ITZ equivalent aperture distribution of CWRB samples under different w/c ratios. Mechanical properties of the Ref group and GO-modified CWRB specimens: (**c**) tensile strength and (**d**) Young’s modulus.

An appropriate w/c ratio can amplify the pore-filling and nucleation effect of GO and promote the hydration reaction of CWRB [32]. Relevant studies have shown that the introduction of GO can improve the microstructure of cement hydration products, refine the crystal size, form a denser and more uniform pore structure network, and achieve the goal of enhancing the macro properties of cement-based composites [33]. To clarify the microscopic enhancement effect of GO on the ITZ in CWRB under different w/c ratios, we combined the metal intrusion technique with BSE images. Compared with traditional Scanning Electron Microscope (SEM) images, BSE images show stronger contrast, higher sensitivity, and significant composition differences in the identification of fine features and pore edge interfaces [34]. Under different w/c ratios, ITZ pore structure color mapping images and measured equivalent pore size distributions of groups Ref and GO were calculated, as shown in Figure 1a,b. It can be seen from Figure 1a,b that when the pore throat is set to 1.54 µm, the pores in the Ref group have good connectivity, and the pore size is distributed between 0 and 19.1 µm. However, most of the pores in the GO group remained independently distributed, with pore sizes ranging from 0 to 17.3 µm, a relative decrease of 9.4%. The color mapping distribution of the aperture in the Ref group showed a trend of diffusion from a small area of dark blue to a large area of green. By comparing the peak pore metal intrusion of the eight groups of samples, it was found that the maximum pore metal intrusion at 13.5 µm in the Ref group was 0.054 mL/g, and that at 9.4 µm in the GO group was 0.04 mL/g. After adding GO, the peak pore diameter was reduced by 30.4% and the peak pore volume by 25.93%. This finding suggests that the presence of GO may alter the hydration path of the ITZ to form a denser and more uniform microstructure, thus promoting the transformation of large pores into small ones [35].

However, with the increase in the w/c ratio, the dilution benefit of GO and the change in hydration kinetics inhibited the reinforcing effect of GO on the ITZ in CWRB. Such weakening effect was mainly reflected in the 20.9% increase in pore size and 26.2% increase in peak pore metal intrusion in the ITZ region under 0.6 and 0.7 w/c ratios, as shown in Figure 1b. After adding GO, the ITZ pore structure of CWRB samples was significantly enhanced, especially at 0.4 and 0.5 w/c ratios. By observing and analyzing the pore size distribution and the peak intrusion of pore metals, it was found that the microscopic characteristics of pore structure are correlated with the changing trend of macroscopic mechanical properties of the CWRB experimental material, as shown in Figure 1c,d. This is consistent with previous studies [22], which showed that with the increase in w/c ratio, the tensile strength and Young’s modulus both showed a trend of first increasing and then decreasing. The tensile property is the highest at 0.5 w/c, and the tensile strength and Young’s modulus reach about 2 MPa and 0.8 GPa, respectively. With a high w/c ratio, the GO nucleation effect gradually weakens, and enhancement efficiency decreases mainly for the following two reasons. The initial hydration process of CWRB samples was relatively complete at a high w/c ratio, which limited the hydration enhancement of GO. In addition, a high w/c ratio will prolong the solidification time of CWRB samples, resulting in poor dispersion of GO in alkaline environments. Due to its great surface energy and oxygen-containing functional groups, graphene oxide will condense rapidly in the slurry, resulting in incomplete hydration in local areas [22]. This also explains why the maximum pore size of GO-4 increased by 6.8% compared with the Ref-4, and the peak pore metal intrusion occurred near the large pore size of 17.3 µm. At a suitable w/c = 0.5, the maximum pore size was reduced from 14.74 µm to 12.69 µm thanks to GO enhancement, and the maximum pore size near 8 µm was reduced by 90.8%. This is because, at the right w/c, GO can be used as a refinement template for the hydration reaction, promoting the growth of C-S-H gel and producing a finer microstructure. In addition, GO’s “wall-like” geometry further creates a pore-filling effect, helping divide the pores in the adhesive into smaller ones without the use of additional hydration products. In summary, GO can refine the microscopic pore structure of CWRB samples by reducing the accumulated porosity inside the CWRB matrix, filling the pore throats of the connecting pores, and densifying high-porosity areas within the transition zone of the interface.

### 3.2. Pore Throat Characterization

**Figure 2:**
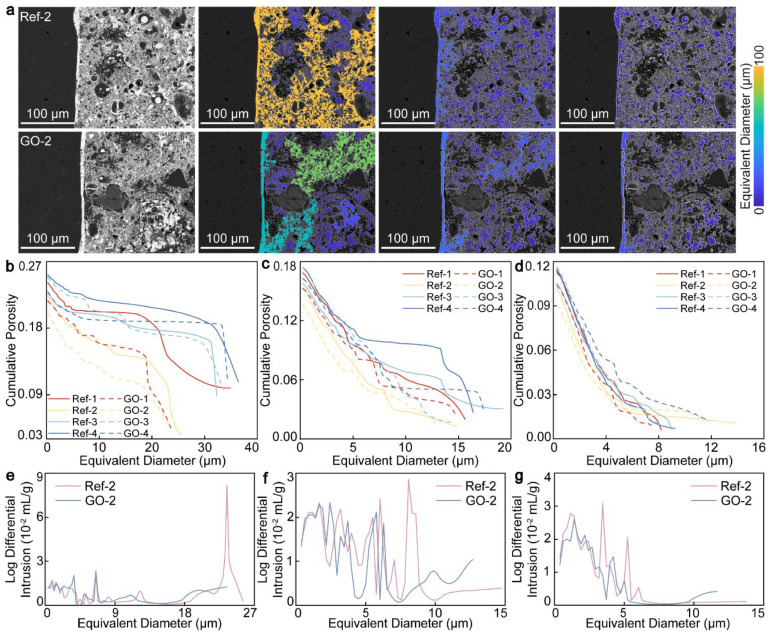
(**a**) The color distribution changes in the ITZ aperture when the pore throat threshold is set to 1.03 µm, 1.54 µm, and 2.05 µm. The relationship between cumulative porosity and equivalent pore size of the ITZ under different pore throats of (**b**) 1.03 µm, (**c**) 1.54 µm, and (**d**) 2.05 µm. The measured equivalent aperture distributions of the ITZ under different pore throats are (**e**) 1.03 µm, (**f**) 1.54 µm, and (**g**) 2.05 µm.

The pore throat structure determines the migration characteristics of the liquid inside the material, which has a great influence on the permeability and durability of the material [10,27]. By studying the pore throat structure of CWRB and using GO to optimize the pore size distribution in the ITZ (reducing harmful coarse pores and increasing beneficial micropores), not only the physical and mechanical properties of the material are improved but also freeze–thaw damage, salt-out erosion, and cracking are prevented [36]. In this section, the effects of GO on the ITZ of CWRB and the pore throat characteristics of the surrounding matrix are analyzed by setting the projection throats as 1.03 µm, 1.54 µm, and 2.05 µm, respectively, and further obtaining BSE images of different pore throats [27]. As shown in Figure 2a, when the projection throat is set to 1.03 µm, most of the pores in ITZ show green and orange-yellow connectivity. With increased pore throat threshold, macropores are gradually refined, showing more independent dark blue micropores. As observed in Figure 2b–d, the cumulative porosity of the ITZ decreases sharply, from 20.05–25.21% at 1.03 µm to 9.01–11.65% at 2.05 µm, and the porosity decreases by 53.5–53.8%. The maximum pore equivalent diameter in the ITZ is reduced by 62.1% from 36.4 µm to 13.8 µm. By changing the threshold value set by the pore throat connection, it was found that with the further increase in the threshold value (1.54 µm to 2.05 µm), the maximum pore equivalent diameter only decreased by 28.1%. The variation range of porosity was greatly narrowed, and the porosity of the eight groups of CWRB samples fluctuated up and down by 2.64%. This significant change demonstrates the presence of a large number of pore throats (1.03 µm–1.54 µm) in the ITZ. As the pore throat size increases to 2.05 µm, the pore throat of 1.03 µm–1.54 µm is no longer recognized as a pore, resulting in a large number of pores being refined into micropores. The change process is shown in Figure 2a.

When the threshold value of pore throat mapping is higher, more pores are actively closed and more connected pores are actively differentiated into micropores in the ITZ, which restricts the optimization effect of GO on the pore network. Therefore, cumulative porosity and equivalent pore size distribution with a calculated pore throat threshold of 1.03 µm were selected for analysis and research, as shown in Figure 2b. When the w/c ratio is 0.5, GO has the best enhancement effect on the ITZ, and the lowest porosity is 20.05%, which is 10.7% lower than that of the control group. According to the measured equivalent aperture distribution curve in Figure 2e, it is mainly reflected that the peak aperture of 23.5 µm in the ITZ group GO-2 is significantly reduced, which is 84.6% lower than that of Ref-2. GO present in the ITZ can form hydrogen bonds with hydroxyl groups (-OH) on the surfaces of the hydration products C-S-H and calcium hydroxide (C-H), thus enabling the nanosheets to promote more robust hydration behavior of CWRB during nucleation. The ITZ enhancement feature of GO at a w/c ratio of 0.5 is mainly reflected in promoting the optimization of large pore sizes (23.5 µm) to small pore sizes (6.5 µm, 3.7 µm). As shown in Figure 2e–g, the regularity of the peak aperture was studied based on the change of the threshold setting of the pore throat. The calculation results show that the peak value of the equivalent aperture is refined to 8.07 µm with the increase in the pore throat, and then further refined to 3.5 µm and 5.3 µm. The thinning characteristics of pore size verify that pore throat can reduce the discrete distribution of pore size and enhance the uniformity and regularity of pore sizes. The w/c ratio also plays a significant role in the pore structure characteristics, which has a great influence on the porosity and pore size. It can be observed from Figure 2b–d that with the increase in w/c ratio, the change in porosity is consistent with the changing trend of measured mechanical test results, showing a general trend of first increasing and then decreasing. The porosity of the Ref-4 group was up to 25.21%, which increased by 25.7% compared with that of the GO-2 group. However, with the increase in the pore throat threshold, GO enhancement was limited at 0.7 w/c ratio and even hindered the pores in the ITZ region for GO-4 group samples at the 2.05 µm pore throat. The main reason is that GO is easy to reaggregate and has poor dispersibility under a high w/c ratio [37]. Secondly, the interface binding force between GO and the ITZ is weak, and GO cannot fully complete the modification process of the ITZ [38]. In this section, through the changes in the pore throat threshold, it is calculated that the ITZ benefits from the nucleation and pore-filling effects of GO, and the optimization of the pore throat is mainly concentrated in the range of 1.03 µm to 1.54 µm. Due to the limited enhancement effect of GO, the pore throat thinning effect of 1.54 µm to 2.05 µm is not significant, and GO is not sufficient to better hinder the pore throat connectivity. On the contrary, with the increase in w/c ratio, GO agglomeration leads to insufficient hydration and pore size optimization, which brings certain side effects to the pore structure. Therefore, when GO is used as a cement-based material modifier, the w/c ratio should be properly controlled to ensure that GO can be evenly dispersed and well combined with the ITZ interface to play its microscale nano-enhancement role [38]. The study of the pore throat structure is helpful in establishing the relationship between microstructure and macroscopic properties, which is of great significance for developing new high-performance cement-based materials.

### 3.3. Pore Profile Characterization

Porosity is an inherent microstructure characteristic of gelled materials. In addition to the pore size distribution, porosity, and pore throat connectivity, the pore profile in the ITZ also greatly influences the permeability and durability of CWRB specimens [39]. In this study, field metal intrusion pores were combined with BSE images to improve image resolution and contrast and obtain a clear view of pore profiles. The concepts of circularity, solidity, and aspect ratio proposed by Chen et al. were used to qualitatively study the shape of pores [40].

**Figure 3:**
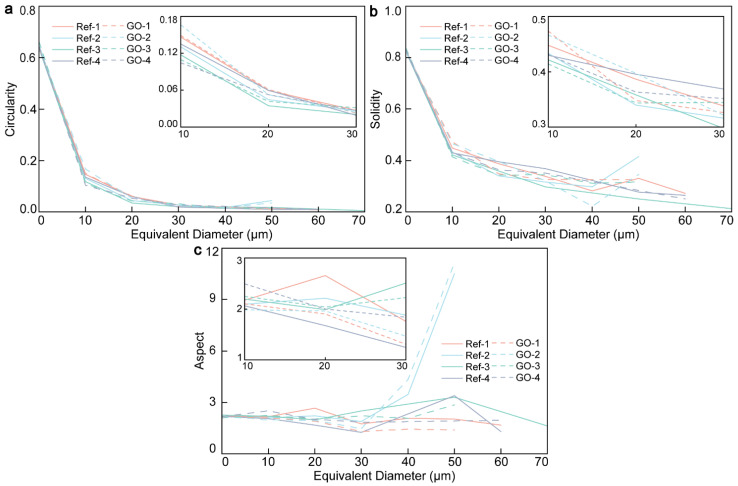
(**a**) Circularity, (**b**) solidity, and (**c**) aspect ratio of the ITZ pore structure in the CWRB sample.

Pore circularity describes the shape characteristics of the pore edge; specifically, it measures the closeness of the pore edge to the ideal circle. Circularity is a dimensionless index commonly used to quantify the geometry of pores [40]. Circularity is usually defined as the ratio of the actual circumference of a void to the circumference of its equivalent circle, which is a circle with the same area as the void. For a perfect circular pore, the circumference P and the area A satisfy the relation P = 2πr and A = πr^2^, then the circularity of the circular pore is 1. For noncircular pores, the roundness is less than 1. The closer the roundness value is to 1, the closer the pore shape is to the circle. The smaller the roundness value, the more irregular the pore shape. The roundness of the pore is an important parameter to evaluate the shape of the pore. The roundness can be obtained by calculating the area and circumference of the pore. This parameter is of great significance in the study of cement-based materials and can be used to evaluate the mechanical properties and durability of materials. The mathematical calculation formula of circularity can be expressed as [40]:

(1) $$C=4\pi \frac{area}{p^{2}}$$

where *C* represents circularity and p represents the circumference of the hole. When *C* = 1, the hole is circular [40]. When the pore shape is curved or the ratio of width to height is large, the *C* value decreases. The porosity roundness has a double effect on the properties of cement-based materials. On the one hand, a higher porosity roundness can reduce the stress concentration and improve the cracking resistance of the material. On the other hand, excessive porosity roundness may lead to increased connectivity between pores, which increases the permeability of the material and reduces its durability. The closer the circularity value is to 1, the better the permeability and fluidity of the material, but a higher circularity value will lead to stress concentration and reduce the mechanical strength and durability of the material [41]. According to the circularity curve in Figure 3a, the w/c ratio has little influence on the circularity of 0–10 µm micropores and large pores above 30 µm, and the curves show a high degree of consistency. GO-4 and Ref-4 with a w/c ratio of 0.7 reached their minimum values at 10 µm and 30 µm, respectively. At 10 µm, the circle’s value decreased by 22.7% thanks to the crack-bridging effect of graphene oxide. In contrast, the circularity of GO-2 reaches its maximum value at 10 µm, increasing by 24.8% compared with the circularity of Ref-2 and decreasing as the aperture increases. The calculation results of the circularity of the transition hole show that the w/c ratio can affect the permeability of the material to some extent. At the same time, GO has a filling effect on macropores, which can split the micro-macropore structure into irregular micropores.

Pore solidity is another important geometric characteristic parameter, which is used to describe the compactness of pore shape. Solidity is defined as the ratio of the actual area of the pore to the minimum convex area. The degree of hydration of cement-based materials can be evaluated by the degree of solidity. Such pore parameters will indirectly affect the mechanical properties and durability of cement-based materials. The solidity index was used to calculate the hydration degree around ITZ and the nucleation effect enhanced by GO [40], and the results are shown in Figure 3b. With the hydration reaction, the solid *S* value of the pore will decrease. The mathematical calculation formula of *S* value can be expressed as [40]:

(2) $$S=\frac{area}{convecx\;area}$$

From the calculation results of 10–30 µm transition holes, the solid degree of CWRB in the four groups after adding GO showed a sharp decline, decreasing by 31.01%, 31.07%, 16.85%, and 18.46%, respectively, which proved that the addition of GO can effectively promote the hydration of cement under the appropriate w/c ratio. With the increase in equivalent diameter, the GO enhancement effect of GO-2 at 40 µm and 50 µm was significant, and the solidity of GO-2 was reduced by 25.3% and 16.9%, respectively, compared with that of Ref-2. With the increase in the w/c ratio, the transition hole fixation curve of the GO-4 group at 0.7 w/c ratios is lower than that of Ref-4. When extended to large pore diameter, the two curves tend to coincide after the equivalent diameter of 30 µm, and the optimization effect of GO is not significant. The changing trend of the S value indicates that GO can form more nucleation sites around the ITZ and promote the hydration process of CWRB. Still, a higher w/c ratio will weaken the enhancement effect of GO.

The aspect ratio, a pore profile parameter, is also closely related to mechanical properties, permeability, and durability. The pore-shape aspect ratio is a descriptor obtained by fitting the pore profile with an ellipse. The mathematical formula for calculating the aspect ratio can be expressed as [40]:

(3) $$A=\frac{L_{major\;axis}}{L_{minor\;axis}}$$

*A* is the aspect ratio, and $L_{major\;axis}$ and $L_{minor\;axis}$ are the long and short axes of the pore profile fitting ellipse, respectively [40]. Pores with a high aspect ratio (slender pores) can become weak links in the material, not only causing the material to be prone to cracks when bearing loads but also increasing the resistance of fluid flow and developing a path for crack expansion [42]. According to the calculation in Figure 3c, the aspect ratio of GO-1 is the lowest, and the value fluctuates between 1.4 and 2.2. After GO was added, the aspect ratio curves decreased by 28.5%, 25.6%, 30.6%, and 31.1% at the equivalent aperture of 10 µm, 20 µm, 30 µm, and 40 µm, respectively. This is because CWRB particles are closer together at low w/c ratios and GO bridges more significantly and hydrates more fully. However, the length aspect ratio curve of GO-2 with an equivalent diameter of 50 µm has a large peak, with an aspect ratio as high as 11.13, an increase of 7.94-fold compared with GO-1. It is speculated that this phenomenon may be due to incomplete regional hydration or the agglomeration of GO near the ITZ. The trend in the aspect ratio indicates that GO is more efficient when the w/c ratio is low. By optimizing the aspect ratio, we can improve the cracking resistance, permeability, and durability of the material.

### 3.4. GO Enhancements in the ITZ of CWRB Based on Deep Learning

**Figure 4:**
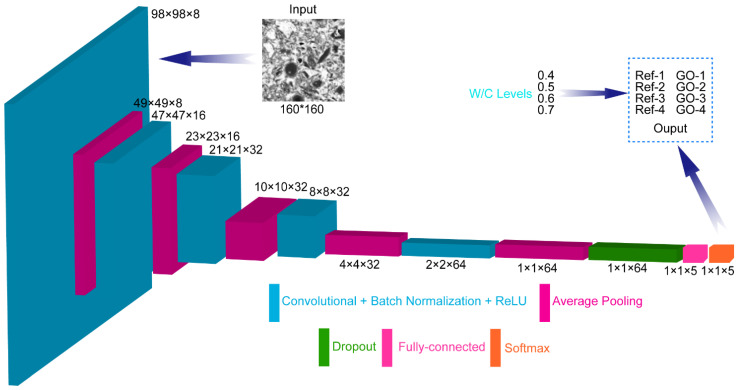
CNN architecture design.

In this section, we use the CNN algorithm to classify and identify ITZ regions of CWRB under different w/c ratios. The BSE image dataset contains four categories, which are 0.4, 0.5, 0.6, and 0.7 w/c ratios with or without GO enhancement. The framework of CNN architecture is shown in Figure 4. The main modules include the convolution layer, normalization layer, pooling layer, activation layer, and output layer. Each class contains thousands of training sets after cropping and rotating and sets with a certain number of validation set and test set images. The number of datasets is achieved by controlling the size of the captured images, the size of the crop area, and the rotation angle, ultimately no fewer than 2000 learned images. The partitioning of the dataset is achieved primarily through the splitEachLabel function in the code. This function is used to randomly segment data from each category in a specified proportion to create training sets, validation sets, and test sets. First, a new dataset is created by randomly selecting 1000 samples for each category from the original dataset. Then the data are randomly divided again according to the proportion of 80%, 10%, and 10%, respectively, as the training set, verification set, and test set. This can not only ensure the randomness of the data but also avoid the phenomenon of overfitting. The filter size for all convolutional layers is set to 3 × 3 with a step length of 1. The filter size for all average pooling layers is set to 2 × 2 step size to 2 [43]. The small batch size, initial learning rate, learning rate attenuation factor, and learning rate attenuation period are set to 500, 0.001, 0.8, and 10, respectively, with specific structures and parameters consistent with those previously reported. Deep learning showed an advantage in extracting representative features, suggesting that higher-order information such as spatial correlation should be a more important factor in cement microstructure.

**Figure 5:**
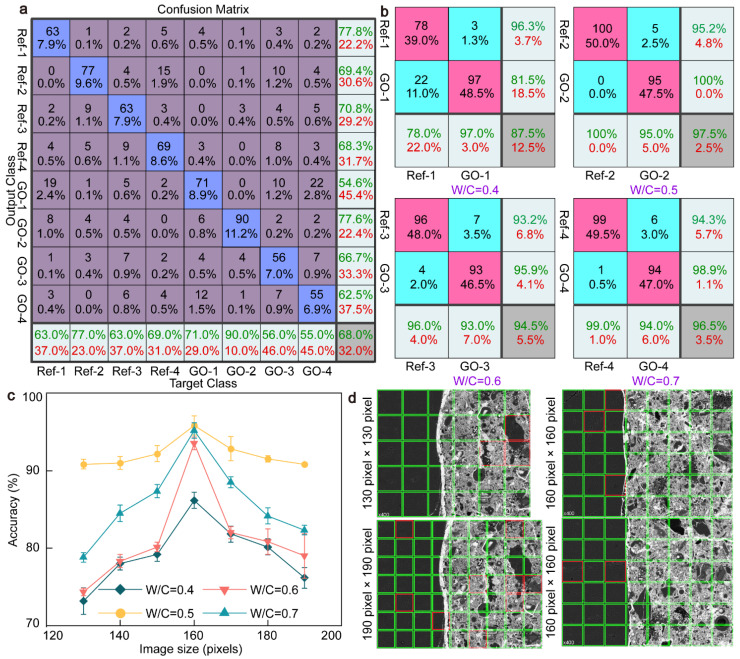
The calculated confusion matrix shows (**a**) the image recognition accuracy at different w/c when the image size is 160 pixels; (**b**) the image recognition accuracy at the same w/c; (**c**) the recognition accuracy of four groups of CWRB samples by a CNN algorithm under different training image sizes; and (**d**) typical examples of image representation precision maps trained at 130, 160, and 190 pixels.

High recognition accuracy is the key to locating micro/nano-modification features and GO quantitative enhancement of the ITZ [44]. Therefore, we studied the recognition accuracy of the CNN algorithm for the ITZ under different pixels, as shown in Figure 5. We calculated the average value and error bar of the sample’s third recognition accuracy, as shown in Figure 5c. It is found that there are significant differences in recognition accuracy under different w/c ratios. The average recognition accuracy of images under a 0.5 w/c ratio is as high as 95.8%, 11.3% higher than that under a 0.4 w/c ratio. In the training process, the size of the training image affects the recognition accuracy of the CWRB sample. The recognition accuracy of CNN for the four groups of samples showed a trend of first increasing and then decreasing, and the recognition accuracy was the highest for the image size of 160 × 160 pixels. This is because the image is too small to ensure that the model can capture important features of the details, affecting the recognition accuracy. However, if the image is too large to contain more noise, there will be confusion in recognition and there will be overfitting. Therefore, resizing the image can better accommodate data enhancement techniques and improve the generalization ability of the model. Multi-class image learning together may also lead to lower recognition accuracy, as shown in Figure 5a. At 160 × 160 pixels, the highest output recognition accuracy of eight kinds of image hybrid learning is only 68%. The highest recognition accuracy of group training is shown in Figure 5b, and the accuracy range is up to 97.5%, a relative increase of 43.4%. The reasons for the reduced accuracy of multi-class image learning together include class imbalance, increased confusion, increased risk of overfitting, increased computational complexity, increased difficulty in feature learning, poor data enhancement, and difficulty in optimization [45]. The representation accuracy diagram in Figure 5d directly reflects the wrong parts identified by the algorithm. We selected the output images with a recognition accuracy of a single training area under 130, 160, and 190 pixels, respectively, where the green box represents the correctly identified part and the red box represents the incorrectly identified part. The red and green frame ratio of the four BSE images shows that the recognition accuracy of the training sample area with 160 × 160 pixels is much higher than that of the 130 × 130 and 190 × 190-pixel areas. By analyzing the error identification parts, it can be found that there is a certain amount of unhydrated cement or small pieces of cement with incomplete hydration in these areas, as well as the parts with relatively independent and dispersed pores. The error areas are mainly concentrated in the ITZ in large unhydrated areas and around coarse particles.

This is mainly due to the coarser aggregates in CWRB samples. Because the enhancement effect of GO cannot be reflected in the coarse aggregate, it leads to difficulty in recognizing the coarse aggregate, which limits the recognition accuracy. In addition, the ITZ around coarse particles worsens the pore structure [46]. Due to the limited optimization effect of GO on pores, it is difficult to distinguish between GO enhancement and non-GO enhancement, which ultimately leads to a decrease in recognition accuracy. Therefore, the key to improving the sample recognition accuracy is to master the enhancement characteristics of GO enhancement while obtaining the appropriate image training size.

### 3.5. GO Enhanced ITZ Feature Model Extraction and Visualization

**Figure 6:**
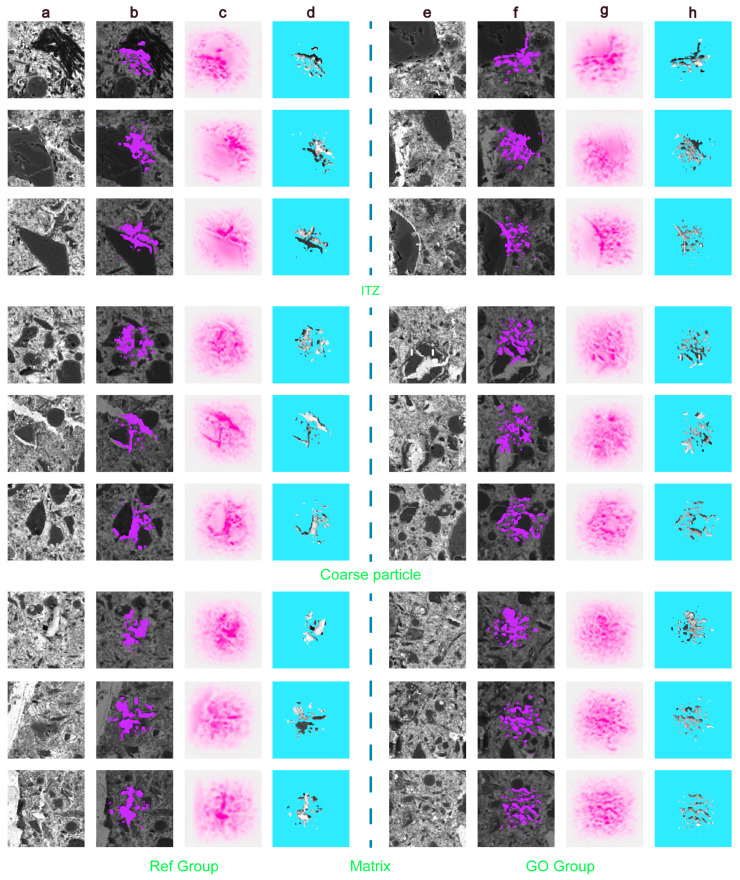
Extraction and visualization of the CWRB sample enhanced feature pattern based on deep learning. (**a**) The Ref group and (**e**) the GO group correctly classified typical BSE images by deep learning. (**b**) DTD visual heat maps of the Ref group and (**f**) the GO group, representing microscale characteristic patterns of BSE images. The correlation between each pixel in the BSE image and the classification decision is represented by a color bar. (**c**) The Ref group and (**g**) the GO group locations of feature regions in training images. Feature regions of (**d**) the Ref group and (**h**) the GO group in CWRB samples extracted from training images.

The deep Taylor decomposition (DTD) algorithm was used to locate the characteristics of coarse particle ITZ [47], matrix pores, and unhydrated ITZ before and after GO enhancement. The core of the DTD method is the interpretation of the classification result of the convolutional neural network as a specific region in the input image. The main stage of extracting the pore structure model by the DTD algorithm is to use thresholding segmentation, region growth, or edge detection to binarize the image and separate the pore and solid parts. Then, the connected regions in the binarization images are marked to extract the geometric features of the pores. Figure 6 shows the microscopic pore structure model of representative parts with an ideal recognition accuracy obtained through deep learning. The algorithm can be used to visualize the pore structure model, classify different types of enhancement characteristics, and intuitively locate the optimization effect of GO on the ITZ pore structure [48]. The DTD visual heat map of the Ref group as a whole presents a large range of flat pink areas, as shown in Figure 6b,f. The blank heat map area mainly contains unhydrated cement or coarse particles, which primarily reflect the hydration effect of this characteristic area. In contrast, after adding GO, the matrix of CWRB and the heat map area of the ITZ show a pink point cell distribution, and the heat map shows an obvious sense of folding. By observing the location of the pore characteristic region in Figure 6c,g, it is found that the addition of GO significantly improves the connectivity of the pore throat. It is mainly reflected in the flat surface and disordered distribution of loose particles in the Ref group, accompanied by good pore connectivity.

Thanks to the enhancement of GO, the extracted feature region presents a dense spider network structure, and the number of pores increases significantly. According to the pore characteristic model in Figure 6d,h, the enhancement effect of GO mainly lies in its ability to narrow pore throats through the pore-filling effect, reduce the connectivity between pores, and transform the clustered patchy pores into disordered scatter distribution. At the same time, by providing more nucleation sites on the surface of coarse particles or near ITZ, the extracted feature regions form a denser network structure of hydration products. The bridge action of GO helps reduce microcracks and pores in the ITZ, improve the microstructure of the ITZ, inhibit the initiation and expansion of coarse cracks, and divide pores into finer and smaller pores. GO also promotes a more robust hydration behavior in the ITZ; the width of ITZ after enhancement is narrower, and the adhesion between coarse particles and the cement matrix is enhanced. In summary, through the pore structure characteristics of the ITZ in the CWRB sample, the differences in pore structure characteristics at the microscopic level can be significantly observed. More importantly, the extracted features can visualize which regions benefit from the enhancement of GO and assess the degree of hydration of CWRB after GO enhancement. To verify that the pore structure model above benefits from the enhancement of GO and estimate the optimization degree of GO, the relevant typical feature regions are selected for calculation and verification.

**Figure 7:**
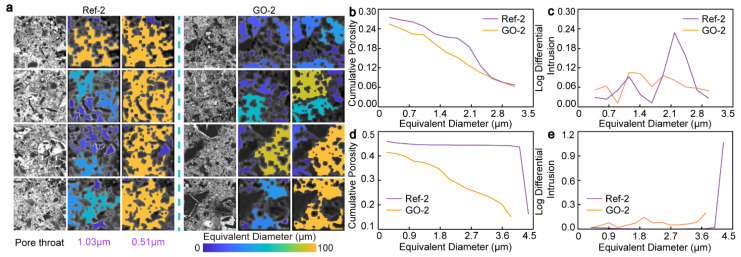
(**a**) Pore color distribution changes in ITZ typical characteristic regions when the pore throat threshold is set to 0.51 µm and 1.03 µm. The relationship between cumulative porosity and equivalent pore size of ITZ under different pore throats is (**b**) 0.51 µm and (**d**) 1.03 µm. The measured equivalent pore size distributions of the ITZ under different pore throats are (**c**) 0.51 µm and (**e**) 1.03 µm.

The ITZ is a vulnerable part of cement-based materials because of its high porosity, large pore size, uneven distribution of hydration products, and low bond strength [49]. By using the DTD algorithm to extract the pore model of CWRB with high accuracy, the above chapters effectively highlight the enhancement effect of GO on the ITZ edge and nearby matrix at the macro level [50]. To establish the relationship between microstructure and macroscopic performance, we selected typical feature regions to locate the optimization effect of GO on the ITZ in CWRB [51]. To clarify the enhancement characteristics of GO, the w/c ratio group with excellent mechanical properties and pore structure after nano-modification was selected to calculate the porosity and measured equivalent pore size distribution, as shown in Figure 7. In this section, four 160 × 160-pixel regions with the most typical characteristics are selected in the Ref-2 and GO-2 groups. The pore size distribution is color-mapped in the BSE images of these small regions at the pore throat of 0.51 µm and 1.03 µm, respectively, to visualize the effect of GO on the microstructure enhancement in the localized ITZ. Compared with the complete BSE image, most of the pore throat connectivity in the ITZ feature region is 0.51 µm–1.03 µm. As shown in Figure 7a, when the pore throat decreases from 1.03 µm to 0.51 µm; most of the independent pores in Ref-2 are connected through the pore throat, while only a small part of the pore throat connectivity exists in the GO-2 group. This phenomenon indicates that GO can refine the pore throat, improve the pore throat connectivity, and adjust the pore throat size distribution to optimize the pore throat structure. Observing the BSE images of selected feature areas, it is found that most of them are concentrated in the coarse particles and the edges of unhydrated areas. This is mainly because ITZ is the most porous on the surface of the coarse particles compared with the gelling matrix, resulting in limited hydration of the cement. Therefore, the enhancement efficiency of graphene oxide is more obvious in the ITZ. The pores in the characteristic region of the Ref-2 group are relatively connected, and the pores show a large area of orange-yellow after mapping. After adding GO, the pores become relatively independent and dispersed, mostly showing dark blue stars or blue patches. To quantitatively analyze and verify the optimization effect of GO on the ITZ typical feature areas, the cumulative porosity and measured equivalent pore size distribution under two kinds of pore throats were calculated, as shown in Figure 7b–e. When the pore throat threshold is set to 1.03 µm, due to the relative independence of pores, the cumulative porosity of the Ref-2 and GO-2 feature regions is 27.5% and 25.6%, respectively, and the porosity decreases by 6.9% after GO is added, as shown in Figure 7b. According to the measured equivalent pore diameter distribution curve under the throat of the pore in Figure 7c, the main function of GO is to inhibit the formation of large pores and narrow them into small pores, which is mainly reflected in that the pore metal intrusion amount of Ref-2 at 2.18 µm increases by 2.77-fold compared with GO-2. With the pore throat threshold reduced to 0.51 µm, due to the enhanced overall pore connectivity, the cumulative porosity of the Ref-2 and GO-2 feature regions is 45.9% and 41.4%, respectively, and the porosity decreases by 9.8% after GO is added, as shown in Figure 7d. The measured equivalent aperture distribution curve under the pore throat is observed in Figure 7e. The maximum equivalent diameter of the GO-2 group is 3.7 µm, which is 11.9% lower than that of the Ref group, which is 4.2 µm. At the same time, the metal intrusion curves of the two pores increase with the increase in the equivalent diameter due to the enhanced connectivity of the pores, and the maximum metal intrusion in the Ref group increases 5.4-fold. The optimization degree of the characteristic region under the two kinds of pore throats is 6.9–9.8%. The reason for the difference in the optimization degree between the two kinds of potholes set at 0.51 µm and 1.03 µm, respectively, is that connectivity occurs when the pore size is larger than the set potholes, while independent distribution occurs when the pore size is smaller. Therefore, the change in the pore throat will affect the degree of optimization, and it can also reflect the optimization efficiency of GO for the pore throat channel of 0.51 µm–1.03 µm. Then, the porosity and metal intrusion amount of the total sample GO-2 and its typical characteristic region in the same pore throat (1.03 µm) were compared. It was found that the porosity of the typical characteristic region was increased by 27.7%, and the pore intrusion increased 1.28-fold. A significant intrusion pore increment verifies that the feature region extracted by the DTD algorithm is located in the ITZ defect region of the CWRB sample. In summary, based on deep learning and combined with DTD algorithm model extraction, it is proved that the metal intrusion characterization method can not only locate specific regions of GO-enhanced ITZ but also calculate the degree of pore optimization of GO in specific regions of cement-based composites.

## 4. Conclusions

In this paper, the metal intrusion experiment combined with BSE images is used to conduct a comprehensive analysis of the measured equivalent pore size distribution, pore throat connectivity, porosity, and pore profile in the ITZ region. Using the CNN algorithm, BSE images before and after GO enhancement under different w/c ratios were learned, recognized, and classified. After obtaining the ideal recognition accuracy, the DTD algorithm was used to extract the microscale model of the ITZ pore structure before and after GO enhancement.

(1) Compared with the Ref group, the pore size of the GO group decreased by 9.4%, and the peak pore intrusion decreased by 25.93%. At a suitable w/c, GO acts as a refinement template for hydration reaction, promoting the growth of C-S-H gel and producing finer microscopic pores. At 0.5 w/c ratio, the maximum pore size of GO-2 decreased by 13.9% compared with Ref-2, and the peak metal intrusion decreased significantly, by 90.8%.
(2) When the pore throat threshold is set to 1.03 µm, the pore structure has good connectivity. With the increase in the pore throat threshold, when it reaches 2.05 µm, the porosity decreases by 53.5–53.8%, and the maximum pore size decreases by 62.1%. However, it was found that the variation range of porosity decreased significantly, between 1.54 µm and 2.03 µm, thanks to the GO enhancement. It is proved that GO can refine the pore throat of the ITZ, and there are a large number of 1.03 µm-1.54 µm pore throats in the ITZ.
(3) The characterization results of the pore profile show that the circularity value decreases by 16.85–31.07% after adding GO. The aspect ratio of the G0-1 group decreased by 25.6–31.1% at different equivalent diameters. At 0.5 w/c ratio, the compactness of the 10 µm transition hole increased by 24.8%. By calculating circularity, solidity, and aspect ratio, it is revealed that GO significantly enhanced the ITZ permeability resistance of CWRB at (0.4–0.5) w/c ratio.
(4) Image learning is used to analyze the microscopic characteristics of the ITZ in CWRB. When the training image size is 41 µm × 41 µm, the classification and recognition accuracy reaches up to 97.5%, a relative improvement of 43.4%. Unhydrated particles and deterioration of ITZ edge pores are the main reasons that limit identification accuracy. Improving the accuracy of the characterization is the key factor in mastering the enhancement characteristics of GO.
(5) The DTD algorithm successfully extracts enhanced features of GO. The calculation results of selected typical feature areas show that GO can enhance ITZ pore weak areas, with the optimization efficiency reaching 6.9–9.8%. The pore size and dispersion of the GO-enhanced pore model are smaller.

## Data Availability

The original contributions presented in this study are included in the article. Further inquiries can be directed to the corresponding author.

## References

[1] Gao Y., Sui H., Yu Z., Wu J., Chen W., Jing H., Ding M., Liu Y. (2022). Industrial graphene oxide-fly ash hybrid for high-performance cemented waste rock backfill. Constr. Build. Mater..

[2] Fang K., Zhang J., Cui L., Haruna S., Li M. (2023). Cost optimization of cemented paste backfill: State-of-the-art review and future perspectives. Miner. Eng..

[3] Aıtcin P. (2003). The durability characteristics of high performance concrete: A review. Cem. Concr. Compos..

[4] Zhan B.J., Xuan D.X., Poon C.S., Scrivener K.L. (2020). Characterization of interfacial transition zone in concrete prepared with carbonated modeled recycled concrete aggregates. Cem. Concr. Res..

[5] Ma H., Tu Y., Yu H., Diao Y., Han W., Zhang M. (2023). Mechanical properties and microstructural characteristics of coral-aggregate-concrete ITZ: Experimental study. J. Build. Eng..

[6] Zhang H., Pei X., Yang Z., Luo S., Yan M., Liu L., Xu Z. (2024). Interfacial design and damage of fiber-reinforced polymer composites/strengthening concrete: A review. J. Mater. Sci..

[7] Lin J., Yao X., de Souza F.B., Sagoe-Crentsil K., Duan W. (2021). Mechanisms of dispersion of nanoparticle-decorated graphene oxide nanosheets in aqueous media: Experimental and molecular dynamics simulation study. Carbon.

[8] Lin J., Shamsaei E., de Souza F.B., Sagoe-Crentsil K., Duan W.H. (2020). Dispersion of graphene oxide–silica nanohybrids in alkaline environment for improving ordinary Portland cement composites. Cem. Concr. Compos..

[9] Birenboim M., Nadiv R., Alatawna A., Buzaglo M., Schahar G., Lee J., Kim G., Peled A., Regev O. (2019). Reinforcement and workability aspects of graphene-oxide-reinforced cement nanocomposites. Compos. Part B Eng..

[10] Cheng Z., Wang J., Hu J., Lu S., Gao Y., Zhang J., Wang S. (2023). Influence of the Graphene Oxide on the Pore-Throat Connection of Cement Waste Rock Backfill. Materials.

[11] Jiang J., Lin J., Tang Z.Q., Lu Z., Wang F., Liu Z. (2024). Decomposition of complex interactions in graphene oxide-superplasticizer-cement composites and proposed guidelines for sample preparation and assessment. Constr. Build. Mater..

[12] Sun H., Ren Z., Ling L., Memon S.A., Ren J., Liu B., Xing F. (2020). Influence of graphene oxide on interfacial transition zone of mortar. J. Nanomater..

[13] Lu D., Qu F., Su Y., Cui K. (2024). Nano-engineered the interfacial transition zone between recycled fine aggregates and paste with graphene oxide for sustainable cement composites. Cem. Concr. Compos..

[14] Yang J., Han S., Wang Q., Wu C., An M., Yu Z., Wang Y., Yan P. (2024). The influence of graphene oxide on the hydration and mechanical properties of cement-based materials with low water-binder ratio. Cem. Concr. Compos..

[15] Panzavolta S., Bracci B., Gualandi C., Focarete M.L., Treossi E., Kouroupis-Agalou K., Rubini K., Bosia F., Brely L., Pugno N.M. (2014). Structural reinforcement and failure analysis in composite nanofibers of graphene oxide and gelatin. Carbon.

[16] Yang H., Cui H., Tang W., Li Z., Han N., Xing F. (2017). A critical review on research progress of graphene/cement based composites. Compos. Part A Appl. Sci. Manuf..

[17] Zhang P., Wang M., Han X., Zheng Y. (2023). A review on properties of cement-based composites doped with graphene. J. Build. Eng..

[18] Phrompet C., Sriwong C., Ruttanapun C. (2019). Mechanical, dielectric, thermal and antibacterial properties of reduced graphene oxide (rGO)-nanosized C3AH6 cement nanocomposites for smart cement-based materials. Compos. Part B Eng..

[19] Regalla S.S. (2024). Effect of nano SiO_2_ on rheology, nucleation seeding, hydration mechanism, mechanical properties and microstructure amelioration of ultra-high-performance concrete. Case Stud. Constr. Mater..

[20] Gao Y., Sui H., Yu Z., Wu J., Chen W., Liu Y. (2023). Cemented waste rock backfill enhancement via fly ash-graphene oxide hybrid under different particle size distribution. Constr. Build. Mater..

[21] Yu Z., Jing H., Gao Y., Xu X., Zhu G., Sun S., Wu J., Liu Y. (2023). Effect of CNT-FA hybrid on the mechanical, permeability and microstructure properties of gangue cemented rockfill. Constr. Build. Mater..

[22] Gao Y., Yu Z., Cheng Z., Chen W., Zhang T., Wu J. (2023). Influence of industrial graphene oxide on tensile behavior of cemented waste rock backfill. Constr. Build. Mater..

[23] Cheng Z., Liu Y., Wu J., Guo X., Chen W., Gao Y. (2023). Graphene oxide-coated fly ash for high performance and low-carbon cementitious composites. J. Mater. Res. Technol..

[24] Zhang J., Scherer G.W. (2011). Comparison of methods for arresting hydration of cement. Cem. Concr. Res..

[25] Liu Y., Chen S.J., Sagoe-Crentsil K., Duan W. (2021). Evolution of tricalcium silicate (C3S) hydration based on image analysis of microstructural observations obtained via Field’s metal intrusion. Mater. Charact..

[26] Willis K.L., Abell A.B., Lange D.A. (1998). Image-based characterization of cement pore structure using wood’s metal intrusion. Cem. Concr. Res..

[27] Shi X., Misch D., Vranjes-Wessely S. (2023). A comprehensive assessment of image processing variability in pore structural investigations: Conventional thresholding vs. machine learning approaches. Gas Sci. Eng..

[28] Zeng H., Qu S., Tian Y., Hu Y., Li Y. (2023). Recent progress on graphene oxide for next-generation concrete: Characterizations, applications and challenges. J. Build. Eng..

[29] Liang M., He S., Gan Y., Zhang H., Chang Z., Schlangen E., Šavija B. (2023). Predicting micromechanical properties of cement paste from backscattered electron (BSE) images by computer vision. Mater. Des..

[30] Lin J., Liu Y., Sui H., Sagoe-Crentsil K., Duan W. (2022). Microstructure of graphene oxide–silica-reinforced OPC composites: Image-based characterization and nano-identification through deep learning. Cem. Concr. Res..

[31] Lin J., Chen S., Wang W., Pathirage C.S.N., Li L., Sagoe-Crentsil K., Duan W. (2021). Transregional spatial correlation revealed by deep learning and implications for material characterisation and reconstruction. Mater. Charact..

[32] Zeng Q., Li K., Fen-Chong T., Dangla P. (2012). Pore structure characterization of cement pastes blended with high-volume fly-ash. Cem. Concr. Res..

[33] Liu C., Huang X., Wu Y.-Y., Deng X., Zheng Z., Xu Z., Hui D. (2021). Advance on the dispersion treatment of graphene oxide and the graphene oxide modified cement-based materials. Nanotechnol. Rev..

[34] Li L., Yang J., Liu W., Ren P. (2023). Overview of the application of quantitative backscattered electron (QBSE) image analysis to characterize the cement-based materials. Constr. Build. Mater..

[35] Zhan P., Xu J., Wang J., Zuo J., He Z. (2022). A review of recycled aggregate concrete modified by nanosilica and graphene oxide: Materials, performances and mechanism. J. Clean. Prod..

[36] Dong H., Gao P., Ye G. (2017). Characterization and comparison of capillary pore structures of digital cement pastes. Mater. Struct..

[37] Liu C., Chen F., Wu Y., Zheng Z., Yang J., Yang B., Yang J., Hui D., Luo Y. (2021). Research progress on individual effect of graphene oxide in cement-based materials and its synergistic effect with other nanomaterials. Nanotechnol. Rev..

[38] Kishore K., Pandey A., Wagri N.K., Saxena A., Patel J., Al-Fakih A. (2023). Technological challenges in nanoparticle-modified geopolymer concrete: A comprehensive review on nanomaterial dispersion, characterization techniques and its mechanical properties. Case Stud. Constr. Mater..

[39] Gao Y., De Schutter G., Ye G., Tan Z., Wu K. (2014). The ITZ microstructure, thickness and porosity in blended cementitious composite: Effects of curing age, water to binder ratio and aggregate content. Compos. Part B Eng..

[40] Chen S.J., Li W.G., Ruan C.K., Sagoe-Crentsil K., Duan W.H. (2017). Pore shape analysis using centrifuge driven metal intrusion: Indication on porosimetry equations, hydration and packing. Constr. Build. Mater..

[41] Al-Hamrani A., Kucukvar M., Alnahhal W., Mahdi E., Onat N.C. (2021). Green concrete for a circular economy: A review on sustainability, durability, and structural properties. Materials.

[42] Tian G., Deng H., Xiao Y. (2022). Correlation Analysis Between Microscopic Pore Parameters and Macroscopic Mechanical Properties of Rock-like Materials from the Perspective of Water-Cement Ratio and Sand-Cement Ratio. Materials.

[43] Wang X., Xiao Y., Yang T., Wang M., Chen Y., Li Z. (2024). Quantitative assessment of cement bridges and voids in cement-stabilized permeable base materials using a mask R-CNN-based CT image segmentation strategy. Mater. Des..

[44] Monteiro P.J., Geng G., Marchon D., Li J., Alapati P., Kurtis K.E., Qomi M.J.A. (2019). Advances in characterizing and understanding the microstructure of cementitious materials. Cem. Concr. Res..

[45] Jiang X., Wang J., Meng Q., Saada M., Cai H. (2023). An adaptive multi-class imbalanced classification framework based on ensemble methods and deep network. Neural Comput. Appl..

[46] Zhang C., Jia Z., Luo Z., Deng Z., Wang Z., Chen C., Zhang Y. (2023). Printability and pore structure of 3D printing low carbon concrete using recycled clay brick powder with various particle features. J. Sustain. Cem.-Based Mater..

[47] Gao Y., Yu Z., Yu S., Sui H., Feng T., Liu Y. (2024). Metal intrusion enhanced deep learning-based high temperature deterioration analysis of rock materials. Eng. Geol..

[48] Clayson I.G., Hewitt D., Hutereau M., Pope T., Slater B. (2020). High throughput methods in the synthesis, characterization, and optimization of porous materials. Adv. Mater..

[49] Mowlaei R., Lin J., de Souza F.B., Fouladi A., Korayem A.H., Shamsaei E., Duan W. (2021). The effects of graphene oxide-silica nanohybrids on the workability, hydration, and mechanical properties of Portland cement paste. Constr. Build. Mater..

[50] Dong S., Wang Y., Ashour A., Han B., Ou J. (2020). Nano/micro-structures and mechanical properties of ultra-high performance concrete incorporating graphene with different lateral sizes. Compos. Part A Appl. Sci. Manuf..

[51] Yu L., Bai S., Guan X. (2023). Effect of graphene oxide on microstructure and micromechanical property of ultra-high performance concrete. Cem. Concr. Compos..

